# Heterotic loci identified for maize kernel traits in two chromosome segment substitution line test populations

**DOI:** 10.1038/s41598-018-29338-1

**Published:** 2018-07-23

**Authors:** Yafei Wang, Xiangge Zhang, Xia Shi, Canran Sun, Jiao Jin, Runmiao Tian, Xiaoyi Wei, Huiling Xie, Zhanyong Guo, Jihua Tang

**Affiliations:** 1 National Key Laboratory of Wheat and Maize Crop Science, Henan Agricultural University, Zhengzhou, 450002 China; 2 Agronomy College, Sichuan Agricultural University, Wenjiang, 611130 China; 3 Xinxiang Academy of Agricultural Sciences, Xinxiang, 453003 China; 4 Hubei Collaborative Innovation Centre for Grain Industry, Yangtze University, Jingzhou, 434025 China

## Abstract

Heterosis has been widely used to increase grain quality and yield, but its genetic mechanism remains unclear. In this study, the genetic basis of heterosis for four maize kernel traits was examined in two test populations constructed using a set of 184 chromosome segment substitution lines (CSSLs) and two inbred lines (Zheng58 and Xun9058) in two environments. 63 and 57 different heterotic loci (HL) were identified for four kernel traits in the CSSLs × Zheng58 and CSSLs × Xun9058 populations, respectively. Of these, nine HL and six HL were identified for four kernel traits in the CSSLs × Zheng58 and CSSLs × Xun9058 populations, at the two locations simultaneously. Comparative analysis of the HL for the four kernel traits identified only 21 HL in the two test populations simultaneously. These results showed that most HL for the four kernel traits differed between the two test populations. The common HL were important loci from the Reid × Tangsipingtou heterotic model, and could be used to predict hybrid performance in maize breeding.

## Introduction

Heterosis is used to describe the superiority of heterozygous genotypes over parental homozygotes with respect to one or more characteristics^1^. Hybrid varieties in many crop species exhibit high fertility rates, improved nutrient quality and content, and an increased resilience under abiotic and biotic stress conditions^2,3^. The use of hybrid seed has significantly increased crop yield, with hybrid rice and maize currently accounting for over 50% of global production^4,5^. A credible approach to estimate hybrid phenotypes resulting from a better comprehension of the genetic basis of heterosis would substantially benefit hybrid breeding programs. In previous studies, dominance, epistasis, and overdominance have been the main genetic models employed to explain heterosis^6–9^. The dominance hypothesis supports the expression at multiple loci of beneficial dominant alleles from both parents that are combined in the hybrid^6,7,10,11^. In contrast, the overdominance hypothesis gives special attention to the presence of loci where the state of heterozygosis surpasses either homozygote^1,12,13^. Last, the interaction among favourable alleles from the given parents at different loci forms the foundation of the epistasis hypothesis^14–18^.

In previous investigations, three types of genetic populations have been used to identify quantitative trait loci (QTL) or heterotic loci (HL). The first population design, which is based on the North Carolina Design III (NCIII), uses F_2_, F_3_, or recombinant inbred lines (RILs) that are backcrossed with their parental lines. Such a design was once used to identify QTLs with overdominant or dominant effects^13,19,20^. Using a design of an immortalised F_2_ (IF_2_) population developed from paired crosses of RILs in rice, Hua *et al*.^16^ detected 44 HL for grain yield and its components in rice, while Tang *et al*.^21^ identified 13 HL for grain yield and its components in maize in the same population. Recently, chromosome segment substitution line (CSSL) backcross populations have been used to identify HL in tomato^22^, rice^23^, and cotton^3^. For instance, Meyer *et al*.^24^ reported a QTL for early stage heterosis related to biomass using testcross hybrids developed from 140 introgression line populations in *Arabidopsis*. Additionally, Shen *et al*.^25^ identified 15 dominance HL for plant height using a test population comprising a set of CSSLs in rice.

Maize has long served as a model species for dichotomising the genetic foundation of heterosis^9^. Despite an extensive and dramatic history of success, a striking discordance still exists, especially in maize, between the widespread agricultural utilisation of hybrid vigour and acknowledging the basis of heterosis^5,8^. Such disharmony blocks the effective exploitation of heterosis. The production of new hybrids is time-consuming^26^, so a better understanding of the underlying genetic basis of heterosis would improve the reliability of predicting hybrid phenotypes for use in hybrid breeding programs.

Grain yield, a complicated trait comprising several major components in different crops, is affected by many genetic and non-genetic factors. In maize, kernels per row, row number, and 100-kernel weight are the three major components of grain yield, with 100-kernel weight showing lower heterosis than the other two^21^. Kernel weight also consists of three secondary traits: kernel length, kernel width, and kernel thickness. Kernel weight and size are characterised as key components of grain yield in different hybrids and their parents^27,28^, and several reports have suggested that kernel length, width, and depth greatly influence kernel weight^29–31^. In the genetic basis of kernel weight and its related traits, several QTL mapping studies have been conducted for maize^21,32–34^, and the QTL for secondary traits of kernel weight, including kernel length, width, depth or thickness, volume, and ratio have also been identified in previous studies^35,36^.

For the heterosis of kernel weight and its related traits, Tang *et al*.^21^ identified two HL for 100-kernel weight in maize, and Wei *et al*.^37^ also reported five HL for 100-kernel weight using a single segment substitution lines testcross population. However, the heterosis of secondary traits of kernel weight is unclear. In this study, we dissected the genetic basis of heterosis for four kernel traits using two test populations constructed from a CSSL population and two test inbred lines, Zheng58 and Xun9058. The HL for the measured traits were identified by comparing single test crosses with the corresponding control hybrid (CK). The objectives of this study were to: (1) detect HL underlying the heterosis for kernel traits, and (2) analyse the genetic basis of heterosis for kernel traits in maize. These HL associated with kernel traits and their associated molecular markers may be used to predict hybrid performance in future maize breeding experiments.

## Results

### Performance of kernel traits and its mid-parent heterosis in the test populations

Average kernel lengths of the CSSLs × Zheng58 population were 10.21 mm and 10.62 mm at Changge and Hebi locations, respectively (Table 1), with corresponding mid-parent heterosis values of 12.31% and 11.46%. These values at the two locations were similar to those of the hybrid Zheng58 × lx9801. Mean kernel widths for the hybrid Zheng58 × lx9801 were 9.23 mm and 9.60 mm at the two locations, with mid-parent heterosis values of 7.88% and 6.27%, respectively. The CSSLs × Zheng58 population mean kernel width was similar to that of the control hybrid at the two locations. Mid-parent heterosis values for kernel width at the two locations were 8.46% and 7.69%, respectively. Regarding kernel thickness, average values of the CSSLs × Zheng58 population were 4.47 mm and 4.59 mm at the two locations, with mid-parent heterosis values of −9.32% and −4.09%, respectively. These test population values were similar to those of the control hybrid. The mean 100-kernel weights for the population were 30.23 g and 34.86 g at the two locations, with 18.61% and 24.34% mid-parent heterosis, respectively.

**Table 1:** Performance of kernel traits in CSSLs × Zheng58 and CSSLs × Xun9058 populations.

Locations	Traits	Parents		Zheng58 × lx9801		CSSLs × Zheng58			
		lx9801	Zheng58	Mean	Mid-parent heterosis (%)	Mean	Mid-parent heterosis (%)	Ske.	Kur.
Changge	KL(mm)	8.93 ± 0.08	9.36 ± 0.02	10.27 ± 0.03	12.28	10.21 ± 0.04	12.31	−0.44	0.19
	KW(mm)	8.50 ± 0.03	8.62 ± 0.07	9.23 ± 0.01	7.88	9.10 ± 0.04	8.46	0.12	−0.68
	KT(mm)	4.57 ± 0.02	5.29 ± 0.04	4.48 ± 0.03	−9.22	4.47 ± 0.01	−9.32	−0.48	0.36
	HKW(g)	21.24 ± 0.19	28.95 ± 0.71	29.75 ± 0.48	18.56	30.23 ± 0.17	18.61	−0.09	−0.57
Hebi	KL(mm)	9.21 ± 0.05	9.76 ± 0.04	10.58 ± 0.02	11.58	10.62 ± 0.06	11.46	−0.15	0.39
	KW(mm)	9.13 ± 0.03	8.93 ± 0.04	9.60 ± 0.04	6.27	9.66 ± 0.06	7.69	0.37	−0.21
	KT(mm)	4.57 ± 0.01	5.14 ± 0.06	4.66 ± 0.04	−3.99	4.59 ± 0.01	−4.09	−0.31	−0.30
	HKW(g)	24.97 ± 1.24	30.7 ± 1.13	34.54 ± 0.42	24.07	34.86 ± 0.18	24.34	−0.14	0.86
**Locations**	**Traits**	**Parents**		**Xun9058 × lx9801**		**CSSLs × Xun9058**			
		**Xun9058**	**CSSLs**	**Mean**	**Mid-parent heterosis (%)**	**Mean**	**Mid-parent heterosis (%)**	**Ske**	**kur**
Changge	KL(mm)	9.80 ± 0.04	8.92 ± 0.06	10 ± 0.03	6.78	9.94 ± 0.03	6.2	−0.16	0.01
	KW(mm)	8.55 ± 0.02	8.90 ± 0.05	9.1 ± 0.02	6.74	9.06 ± 0.03	3.84	−0.51	0.49
	KT(mm)	5.29 ± 0.06	4.58 ± 0.04	4.37 ± 0.01	−11.35	4.41 ± 0.01	−10.64	0.02	0.24
	HKW(g)	24.17 ± 0.56	21.22 ± 0.25	24.91 ± 0.47	9.71	25.73 ± 0.25	13.37	0.01	0.60
Hebi	KL(mm)	10.77 ± 0.06	9.23 ± 0.06	10.69 ± 0.04	7.01	10.63 ± 0.08	6.91	−0.47	0.85
	KW(mm)	8.93 ± 0.03	8.90 ± 0.07	10.01 ± 0.03	10.82	9.87 ± 0.09	12.30	−0.18	−0.62
	KT(mm)	5.14 ± 0.07	4.58 ± 0.03	4.57 ± 0.04	−5.85	4.54 ± 0.02	−5.93	0.46	0.47
	HKW(g)	30.23 ± 0.82	24.85 ± 0.29	34.35 ± 0.67	24.46	34.41 ± 0.29	24.73	0.01	−0.01

KL, kernel length; KW, kernel width; KT, kernel thickness; HKW, 100-kernel weight; ± , standard deviation.

Mean kernel lengths for the hybrid Xun9058 × lx9801 were 10.00 mm and 10.69 mm, with 6.78% and 7.01% mid-parent heterosis values at the two locations, respectively. The CSSLs × Xun9058 population had the closest kernel length and mid-parent heterosis to its control hybrid. Regarding kernel width, average values for the CSSLs × Xun9058 population were 9.06 mm and 9.87 mm, with 3.84% and 12.30% mid-parent heterosis at the two locations, respectively. The CSSLs × Xun9058 population had the closest values of kernel thickness and its mid-parent heterosis to the control hybrid. The mean 100-kernel weights for the CSSLs × Xun9058 population were 25.73 g and 34.41 g, and mid-parent heterosis values were 13.37% and 24.73% at the two locations, respectively.

The four kernel traits of the two test populations exhibited significant variation between locations and genotypes (*P* < 0.01; Table 2), and only kernel thickness showed significant location × genotype interaction variation at the *P* < 0.05 level. The heritability (*H*^2^_*B*_) of kernel length, width, thickness, and 100-kernel weight was 73.50%, 73.18%, 78.58%, and 67.62%, respectively, in the CSSLs × Zheng58 population, with a relative lower heritability of the four measured traits (65.22%, 64.85%, 67.35%, and 64.89%); kernel thickness showed the highest heritability and negative mid-parent heterosis of all four kernel traits.

**Table 2:** Analysis of variance for the four kernel traits in CSSLs × Zheng58 and CSSLs × Xun9058 populations.

Traits	Populations	CSSLs × Zheng58					CSSLs × Xun9058				
		DF	SS	MS	F	P	DF	SS	MS	F	P
Kernel length	Location	1	40.42	40.42^**^	289.29	<0.0001	1	89.11	89.11^**^	527.35	<0.0001
	Repetition	4	1.77	0.44	3.17	0.014	4	0.76	0.19	1.13	0.34
	Genotype	175	43.30	0.25^**^	1.77	<0.0001	164	41.29	0.25^**^	1.49	0.0006
	Location × Genotype	171	28.62	0.17	1.20	0.07	159	26.81	0.17	1.00	0.49
	Error	507	70.84	0.14			466	78.74	0.17		
	Heritability (*H*^*2*^_*B*_)	73.50%					65.22%				
Kernel width	Location	1	69.68	69.68^**^	300.37	<0.0001	1	114.95	114.95^**^	393.68	<0.0001
	Repetition	4	9.94	2.49^**^	10.71	<0.0001	4	1.27	0.32	1.09	0.36
	Genotype	175	57.85	0.33^**^	1.43	0.002	164	44.28	0.27^**^	0.93	0.005
	Location × Genotype	171	39.08	0.23	0.99	0.54	159	53.49	0.34	1.15	0.13
	Error	507	117.62	0.23			466	136.07	0.29		
	Heritability (*H*^*2*^_*B*_)	73.18%					64.85%				
Kernel thickness	Location	1	346.67	346.67^**^	97.27	<0.0001	1	338.07	338.07^**^	81.32	<0.0001
	Repetition	4	12.76	3.19	0.89	0.47	4	10.02	2.50	0.60	0.66
	Genotype	175	1291.76	7.38^**^	2.07	<0.0001	164	1539.45	9.39^**^	2.26	<0.0001
	Location × Genotype	174	754.52	4.34^*^	1.22	0.04	164	861.48	5.25^*^	1.26	0.03
	Error	610	2174.06	3.56			572	2377.90	4.16		
	Heritability (*H*^*2*^_*B*_)	78.58%					67.35%				
100-kernel weight	Location	1	3038.61	3038.61^**^	683.33	<0.0001	1	10502.4	10502.4^**^	1654.41	<0.0001
	Repetition	4	280.60	70.15^**^	15.78	<0.0001	4	19.21	4.80	0.76	0.55
	Genotype	175	1210.44	6.92^**^	1.56	0.0002	164	1616.15	9.85^**^	1.55	0.0003
	Location × Genotype	157	719.88	4.59	1.03	0.4	149	1281.23	8.6^*^	1.35	0.01
	Error	404	1796.49	4.45			373	2367.85	6.35		
	Heritability (*H*^*2*^_*B*_)	67.62%					64.89%				

DF, degrees of freedom; SS, Sum of square; MS, mean square; ^*, **^p < 0.05 and *p* < 0.01, respectively.

### Correlations between phenotype and heterosis for the four kernel traits

For the CSSLs × Zheng58 population, kernel length was significantly correlated with kernel width (*P* < 0.01; Table 3), while 100-kernel weight was significantly correlated with kernel length, width, and thickness (*P* < 0.01). The mid-parent heterosis for kernel length was also significantly correlated with kernel width and 100-kernel weight (*P* < 0.01); however, kernel length was negatively correlated with kernel thickness (*P* < 0.01). Additionally, kernel width was significantly correlated with 100-kernel weight (*P* < 0.01).

**Table 3:** Correlation coefficients for four kernel traits in CSSLs × Zheng58 and CSSLs × Xun9058 populations.

Populations	Trait	Kernel length	Kernel width	Kernel thickness	100-kernel weight
CSSLs × Zheng58	Kernellength		0.25^**^	−0.09	0.19^**^
	Kernelwidth	0.25^**^		0.05	0.35^**^
	Kernel thickness	−0.24^**^	0.06		0.27^**^
	100-kernels weight	0.20^**^	0.31^**^	0.04	
CSSLs × Xun9058	Kernellength		0.25^**^	−0.10	0.21^**^
	Kernelwidth	0.16		0.05	0.36^**^
	Kernel thickness	−0.14	0.03		0.27^**^
	100-kernel weight	0.03	0.21^**^	0.16^*^	

Correlation coefficients for phenotype and mid-parent heterosis of each trait are listed above and below the diagonal. ^*, **^p < 0.05 and *p* < 0.01, respectively.

In the CSSLs × Xun9058 population, significant correlations were observed between kernel length and kernel width (*P* < 0.01), and between 100-kernel weight and kernel length, width, and thickness (*P* < 0.01). Regarding mid-parent heterosis, the 100-kernel weight was significantly correlated with kernel width and thickness (*P* < 0.01 and *P* < 0.05, respectively).

### QTL detected for the four kernel traits in the CSSL population

For kernel length, 10 QTL were identified using the average data of each CSSL in the same location over 2 years (Table 4). Among them, the QTL *qKL4* was detected in two locations simultaneously, making a 14.69% and 3.09% phenotypic contribution, and increasing kernel length by 0.13 mm and 0.03 mm at Changge and Hebi, respectively. Nine QTL for kernel width were also identified in the CSSL population at the two locations, and QTL *qKW6b* accounted for −3.90% and −3.83% phenotypic variation at the two locations. Seventeen QTL were identified for kernel thickness, with QTL *qKT3b*, *qKT6d*, and *qKT9b* detected at the two locations simultaneously. For 100-kernel weight, 20 QTL were detected, of which *qHKW6f* accounted for −6.86% and −5.07% phenotypic contribution at the two locations, and QTL *qHKW9b* made contributions of −6.11% and −4.03% in the CSSL population.

**Table 4:** QTL detected for the four kernel traits in the CSSLs population.

Location	Trait	QTL	Bin	Chromosomal region	Additive	Contribution (%)	*P* value
Changge	Kernel length	*qKL1*	1.06	umc1035-umc1335-umc2396	0.13	14.69	0.019
		*qKL2*	2.04	umc2088-umc1485-bnlg1861	−0.02	−2.39	0.036
		*qKL4*	4.01	umc1017-umc1757-umc2280	−0.03	−3.23	0.009
		*qKL6a*	6.05	umc1614-umc2141-umc1805	−0.03	−3.51	0.013
		*qKL9a*	9.02	umc1170-umc1037-umc1033	−0.05	−5.75	0.045
	Kernel width	*qKW1a*	1.06	umc1035-umc1335-umc2396	0.10	12.09	0.009
		*qKW2*	2.04	umc2088-umc1485-bnlg1861	−0.03	−2.92	0.011
		*qKW4*	4.01	umc1017-umc1757-umc2280	0.03	2.97	0.011
		*qKW6a*	6.04	umc2006-umc1614-umc2141	0.03	2.97	0.014
		*qKW6a*	6.05	umc1614-umc2141-umc1805	0.02	2.62	0.035
		*qKW6b*	6.07	umc1433-bnlg1380-bnlg1792	−0.03	−3.90	0.049
	Kernel thickness	*qKT1a*	1.07	umc1356-umc1278-umc1013	−1.80	−3.86	0.040
		*qKT1b*	1.08	bnlg2228-dupssr12-umc2047	−2.13	−4.59	0.006
		*qKT1d*	1.11	umc2047-umc1538-bnlg131	2.21	4.77	0.006
		*qKT2*	2.04	bnlg1064-umc1024-umc1465	−0.54	−1.17	0.030
		*qKT2*	2.04	bnlg1064-umc1024-umc1465	−1.48	−3.19	0.027
		*qKT3a*	3.07	umc1489-umc1825-phi046	−1.51	−3.25	0.017
		*qKT3b*	3.08	umc1844-umc2275-umc2081	−3.55	−7.65	0.004
		*qKT6a*	6.00	phi075-bnlg238	−1.58	−3.41	0.000
		*qKT6d*	6.08	phi123-umc1127	2.08	4.48	0.001
		*qKT7*	7.03	bnlg2271-umc1112-bnlg1805	1.64	3.54	0.003
		*qKT9b*	9.02	umc1170-umc1037-umc1033	−2.14	−4.61	0.009
	100-kernel weight	*qHKW1*	1.03	umc1403-umc1397-bnlg182	−0.71	−3.55	0.007
		*qHKW2b*	2.04	umc2088-umc1485-bnlg1861	−1.41	−7.03	0.031
		*qHKW3*	3.03	umc2259-phi036-umc1495	−1.62	−8.09	0.007
		*qHKW4a*	4.01	umc1017-umc1757-umc2280	1.11	5.56	0.027
		*qHKW5a*	5.00	umc2302-umc1990-umc1482	0.71	3.53	0.024
		*qHKW6f*	6.07	umc1433-bnlg1380-bnlg1792	−1.37	−6.86	0.013
		*qHKW9a*	9.00	umc1037-umc1033-bnlg1082	−1.09	−5.43	0.016
		*qHKW9b*	9.02	umc1170-umc1037-umc1033	−1.22	−6.11	0.002
		*qHKW9c*	9.04	umc1522-umc1492-umc1519	−1.60	−8.02	0.037
		*qHKW9d*	9.06	bnlg1191-umc2345-umc1310	1.94	9.7	0.033
Hebi	Kernel length	*qKL4*	4.01	umc1017-umc1757-umc2280	0.03	3.09	0.049
		*qKL5*	5.00	umc2302-umc1990-umc1482	0.06	5.67	0.018
		*qKL6b*	6.05	bnlg1732-umc1424-umc1296	0.05	5.5	0.012
		*qKL9b*	9.04	umc1492-umc1519-umc1375	0.04	3.78	0.058
		*qKL9c*	9.06	bnlg1191-umc2345-umc1310	0.04	4.38	0.047
		*qKL10*	10.04	umc1336-umc2163-umc2350	0.06	6.36	0.026
	Kernel width	*qKW1b*	1.09	umc2047-umc1538-bnlg131	−0.05	−5.11	0.033
		*qKW3*	3.08	umc1844-umc2275-umc2081	−0.07	−7.57	0.037
		*qKW6b*	6.07	umc1433-bnlg1380-bnlg1792	−0.04	−3.83	0.012
		*qKW9a*	9.04	umc1492-umc1519-umc1375	−0.04	−4.2	0.026
		*qKW9b*	9.06	bnlg1191-umc2345-umc1310	0.06	6.11	0.015
	Kernel thickness	*qKT1b*	1.08	bnlg2228-dupssr12-umc2047	−1.81	−3.95	0.011
		*qKT1c*	1.09	umc2047-umc1538-bnlg131	2.53	5.54	0.005
		*qKT2*	2.04	umc2088-umc1485-bnlg1861	−0.78	−1.7	0.008
		*qKT3b*	3.08	umc1844-umc2275-umc2081	−3.22	−7.05	0.008
		*qKT5*	5.00	umc1496-umc1097-bnlg1006	1.97	4.3	0.002
		*qKT6b*	6.00	umc1178-phi389203-umc2316	−1.26	−2.75	0.002
		*qKT6c*	6.07	umc1433-bnlg1380-bnlg1792	−1.75	−3.84	0.003
		*qKT6d*	6.08	phi123-umc1127	0.87	1.9	0.033
		*qKT9a*	9.00	bnlg1272-bnlg1810-umc1809	2.47	5.4	0.027
		*qKT9b*	9.02	umc1170-umc1037-umc1033	−1.82	−3.97	0.015
		*qKT9c*	9.04	umc1492-umc1519-umc1375	1.11	2.44	0.019
		*qKT9d*	9.05	bnlg1091-bnlg1191-umc2345	2.90	6.34	0.005
	100-kernel weight	*qHKW2a*	2.03	bnlg1064-umc1024-umc1465	−1.55	−4.97	0.038
		*qHKW4b*	4.00	umc1232-phi072-umc1228	0.57	1.82	0.036
		*qHKW5b*	5.05	umc1729-bnlg118-umc1792	1.53	4.91	0.000
		*qHKW6a*	6.02	umc1979-nc009-umc1014	1.21	3.9	0.005
		*qHKW6b*	6.04	umc2006-umc1614-umc2141	0.73	2.34	0.018
		*qHKW6c*	6.04	nc009-umc1014-mmc0523	1.19	3.83	0.027
		*qHKW6d*	6.04	nc012-umc1020-bnlg1732	−1.86	−5.98	0.008
		*qHKW6e*	6.05	bnlg1732-umc1424-umc1296	1.74	5.58	0.000
		*qHKW6f*	6.07	umc1433-bnlg1380-bnlg1792	−1.58	−5.07	0.000
		*qHKW6g*	6.07	phi123-umc1127	0.55	−0.77	0.039
		*qHKW9b*	9.02	umc1170-umc1037-umc1033	−1.26	−4.03	0.005
		*qHKW10*	10.04	umc1336-umc2163-umc2350	0.79	2.53	0.014

### Heterotic loci identified for kernel traits in the CSSLs × Zheng58 population

**Figure 1:**
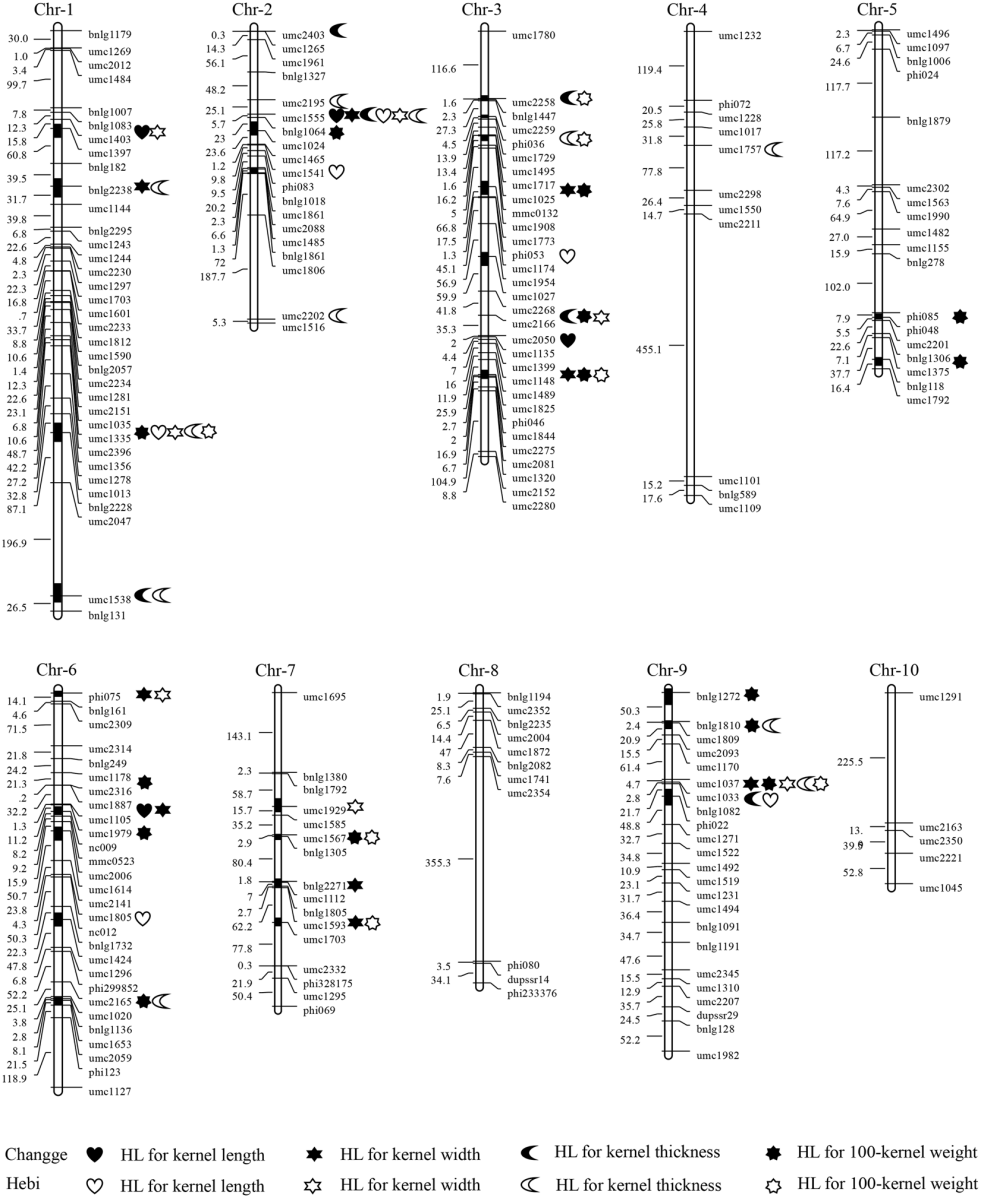
Integrated heterotic loci (HL) on genetic linkage maps for kernel traits in two environments in the CSSLs × Zheng58 population. The heterotic chromosome ranges are represented by black color.

A total of 63 different HL were identified for the four kernel traits in the CSSLs × Zheng58 population at the two locations (Table 5; Fig. 1). Of these, 14 different HL for kernel length were detected in the test populations; *hKL9b*, identified at the two locations simultaneously, had −4.21% and −4.57% control heterosis at Changge and Hebi, respectively.

**Table 5:** Heterotic loci detected for the four kernel traits in the CSSLs × Zheng58 population.

Locations	Traits	HL	Bin	Chromosomal region	Control heterosis (%)	*P* value
Changge	Kernel length	*hKL1b*	1.03	umc1397-bnlg182-bnlg2238	−7.12	0.004
		*hKL1c*	1.06	umc1035-umc1335-umc2396	−7.28	0.028
		*hKL3c*	3.07	umc1135-umc1399-umc1148	−7.28	0.016
		*hKL3d*	3.07	umc1844-umc2275-umc2081	−7.77	0.032
		*hKL4*	4.01	umc1228-umc1017-umc1757	−6.31	0.024
		*hKL5a*	5.06	bnlg278-umc1680-phi085	−6.8	0.045
		*hKL6a*	6.04	mmc0523-umc2006-umc1614	−7.44	0.005
		*hKL7b*	7.03	umc1567-bnlg1305-bnlg2271	6.80	0.045
		*hKL9b*	9.03	bnlg1082-phi022-umc1271	−4.21	0.023
	Kernel width	*hKW1b*	1.04	bnlg182-bnlg2238-umc1144	5.54	0.006
		*hKW1d*	1.08	bnlg2228-dupssr12-umc2047	10.15	0.010
		*hKW2*	2.03	umc2195-umc1555-bnlg1064	5.54	0.028
		*hKW3a*	3.04	umc2259-phi036-umc1495	5.54	0.042
		*hKW3b*	3.05	umc1174-bnlg1035-umc2127	6.27	0.016
		*hKW3d*	3.08	umc1844-umc2275-umc2081	−5.9	0.029
		*hKW3d*	3.08	phi046-umc1844-umc2275	6.27	0.043
		*hKW6b*	6.04	umc1979-nc009-umc1014	6.83	0.014
		*hKW7a*	7.02	bnlg1792-umc1929-umc1585	5.90	0.005
		*hKW7c*	7.03	umc1567-bnlg1305-bnlg2271	5.17	0.026
		*hKW7d*	7.04	umc2332-phi328175-umc1295	8.12	0.036
		*hKW9a*	9.02	umc1037-umc1033-bnlg1082	4.06	0.049
		*hKW9d*	9.06	umc1310-umc2207-dupssr29	4.61	0.040
	Kernel thickness	*hKT1d*	1.11	bnlg2228-dupssr12-umc2047	−5.53	0.024
		*hKT2a*	2.03	umc2195-umc1555-bnlg1064	−3.66	0.026
		*hKT3a*	3.03	phi374118-umc2258-bnlg1447	−4.15	0.021
		*hKT3c*	3.05	phi053-umc1174-bnlg1035	6.05	0.034
		*hKT3d*	3.05	umc2127-umc1954-umc2166	−4.51	0.039
		*hKT5*	5.04	umc2302-umc1990-umc1482	3.43	0.041
		*hKT7b*	7.03	umc1567-bnlg1305-bnlg2271	10.56	0.037
		*hKT8*	8.03	bnlg2082-umc1741-umc2354	5.28	0.015
		*hKT9c*	9.03	bnlg1082-phi022-umc1271	−10.19	0.028
	100-kernel weight	*hHKW1b*	1.04	bnlg182-bnlg2238-umc1144	8.95	0.001
		*hHKW1c*	1.07	umc1335-umc2396-umc1356	7.98	0.005
		*hHKW1c*	1.07	umc1356-umc1278-umc1013	6.81	0.008
		*hHKW1d*	1.08	umc1278-umc1013-bnlg2228	7.39	0.005
		*hHKW2b*	2.03	umc2195-umc1555-bnlg1064	6.81	0.026
		*hHKW3a*	3.03	phi374118-umc2258-bnlg1447	6.81	0.008
		*hHKW3b*	3.04	umc2259-phi036-umc1495	12.06	0.000
		*hHKW3d*	3.05	umc2127-umc1954-umc2166	−13.62	0.002
		*hHKW7b*	7.03	umc1567-bnlg1305-bnlg2271	11.48	0.006
		*hHKW9a*	9.00	phi233376-bnlg1272-bnlg1810	−6.61	0.046
		*hHKW9c*	9.02	umc1170-umc1037-umc1033	12.06	0.035
		*hHKW9d*	9.03	bnlg1082-phi022-umc1271	12.65	0.004
		*hHKW9e*	9.06	umc1310-umc2207-dupssr29	8.56	0.024
		*hHKW10*	10.04	umc2163-umc2350-umc1272	6.23	0.018
Hebi	Kernel length	*hKL1d*	1.08	umc1013-bnlg2228-dupssr12	5.20	0.002
		*hKL2a*	2.03	umc2195-umc1555-bnlg1064	4.88	0.043
		*hKL2b*	2.04	umc2088-umc1485-bnlg1861	7.24	0.001
		*hKL3a*	3.06	umc1593-umc1027-umc2268	4.57	0.002
		*hKL6b*	6.06	bnlg1732-umc1424-umc1296	−4.09	0.003
		*hKL9b*	9.03	bnlg1082-phi022-umc1271	−4.57	0.01
	Kernel width	*hKW1a*	1.03	umc1397-bnlg182-bnlg2238	9.03	0.021
		*hKW1c*	1.07	umc1335-umc2396-umc1356	6.94	0.034
		*hKW2*	2.03	umc2195-umc1555-bnlg1064	5.73	0.037
		*hKW3c*	3.05	umc1954-umc2166-umc1593	−5.90	0.027
		*hKW6a*	6.00	phi075-bnlg238	5.21	0.038
		*hKW7a*	7.02	bnlg1792-umc1929-umc1585	4.51	0.023
		*hKW9a*	9.02	umc1037-umc1033-bnlg1082	10.76	0.003
	Kernel thickness	*hKT1a*	1.04	bnlg182-bnlg2238-umc1144	−5.06	0.034
		*hKT1c*	1.08	umc1013-bnlg2228-dupssr12	6.55	0.047
		*hKT1d*	1.11	umc2047-umc1538-bnlg131	−4.09	0.023
		*hKT2a*	2.03	bnlg1327-umc2195-umc1555	−4.89	0.010
		*hKT2a*	2.03	umc2195-umc1555-bnlg1064	−14.08	0.017
		*hKT2b*	2.08	umc1806-umc2202-umc1516	−5.71	0.036
		*hKT3b*	3.04	umc1717-umc1025-mmc0132	−5.52	0.033
		*hKT4b*	4.03	umc1757-umc2280-umc1550	5.37	0.029
		*hKT6e*	6.07	bnlg1136-umc1653-umc2059	6.42	0.025
		*hKT9a*	9.01	bnlg1272-bnlg1810-umc1809	−7.75	0.025
		*hKT9b*	9.02	umc1170-umc1037-umc1033	6.21	0.001
	100-kernel weight	*hHKW1d*	1.08	umc1013-bnlg2228-dupssr12	5.20	0.043
		*hHKW3a*	3.03	phi374118-umc2258-bnlg1447	6.17	0.014
		*hHKW3b*	3.04	umc2259-phi036-umc1495	10.03	0.039
		*hHKW3f*	3.08	umc1844-umc2275-umc2081	12.6	0.024
		*hHKW7a*	7.02	umc1666-umc1703-umc1433	15.82	0.007
		*hHKW7b*	7.03	umc1567-bnlg1305-bnlg2271	6.49	0.013
		*hHKW9b*	9.02	umc1037-umc1033-bnlg1082	10.99	0.025

HL, heterotic loci.

Sixteen HL for kernel width were detected in the CSSLs × Zheng58 population at the two locations, including *hKW*2, *hKW7a*, and *hKW9a*, which were detected at both locations simultaneously. *hKW*2 increased kernel width by 5.54% and 5.73% compared with the control hybrid at Changge and Hebi, respectively, compared with 5.90% and 4.51% for *hKW7a*, and 4.06% and 10.76% for *hKW9a*.

We identified 17 different HL for kernel thickness in the CSSLs × Zheng58 population at the two locations, including nine detected at Changge and 10 at Hebi. *hKT1d* decreased the kernel thickness by −5.53% and −4.09% compared with the control hybrid, while *hKT*2*a* made −3.66% and −4.89% contributions to heterosis for kernel thickness at Changge and Hebi, respectively.

For 100-kernel weight, 16 HL were detected in the CSSLs × Zheng58 populations at the two locations, including 13 at Changge and seven at Hebi. Four of these, *hHKW1d*, *hHKW3a*, *hHKW3b*, and *hHKW7b* were identified at both locations simultaneously. *hHKW1d* increased the 100-kernel weight by 7.39% and 5.20%, and *hHKW3b* increased it by 12.60% and 10.30% compared with the control hybrid at the two locations, respectively. HL *hHKW7b* made 7.03% and 6.49% contributions to heterosis of the 100-kernel weight at the two locations, respectively.

### Heterotic loci identified for kernel traits in the CSSLs × Xun9058 population

**Figure 2:**
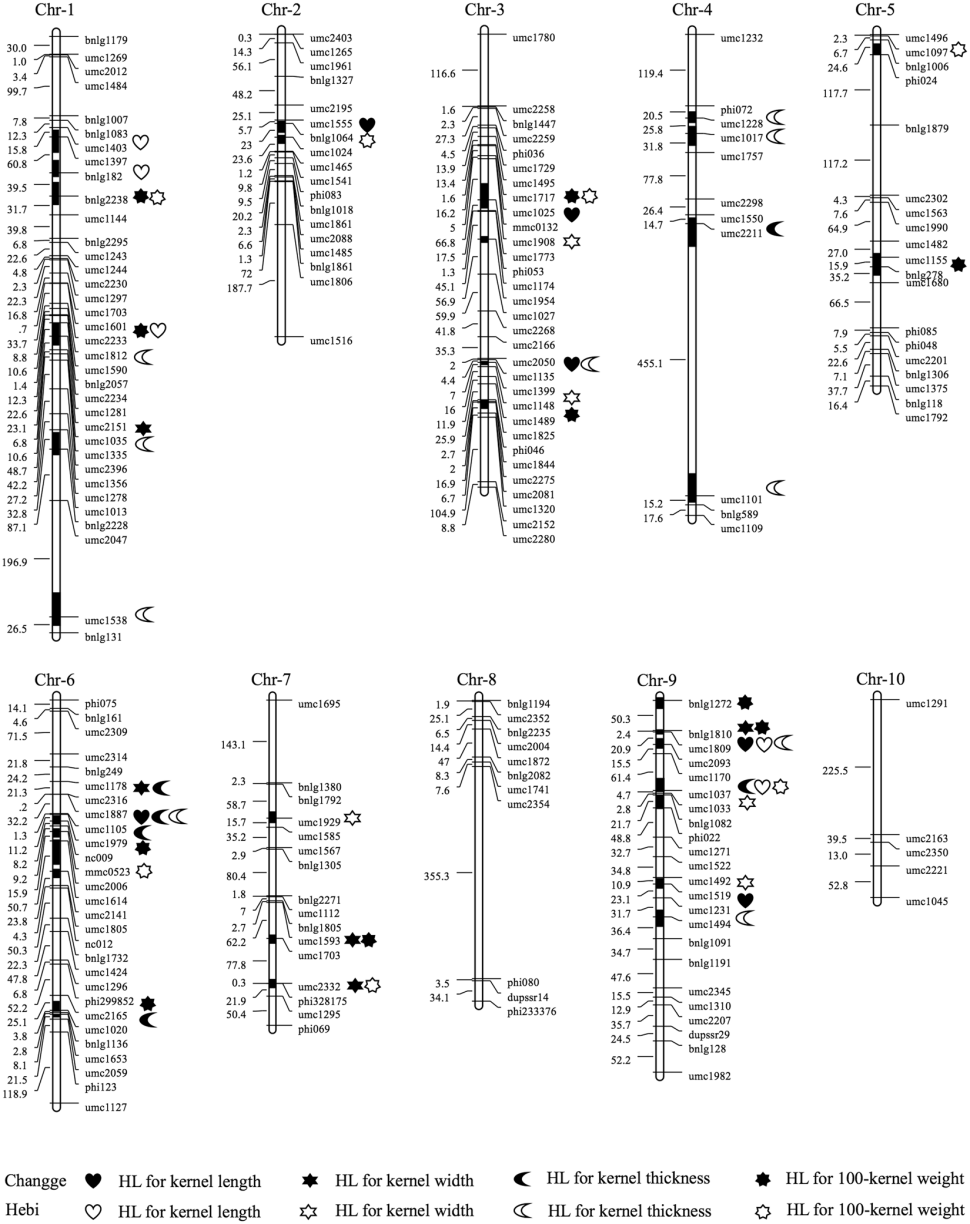
Integrated heterotic loci (HL) on genetic linkage maps for kernel traits in two environments in the CSSLs × Xun9058 population. The heterotic chromosome ranges are represented by black color.

A total of 57 different HL for the four kernel traits were identified in the CSSLs × Xun9058 population at the two locations (Table 6; Fig. 2), including 36 detected at Changge and 26 at Hebi. For kernel length, a total of nine HL were detected in the populations at the two locations. *hKL9a* decreased the kernel length by 7.00% and 3.17% compared with the control hybrid at the two locations, respectively.

**Table 6:** Heterotic loci detected for kernel-related traits in the CSSLs × Xun9058 population.

Locations	Traits	HL	Bin	Chromosomal region	Control heterosis(%)	P value
Changge	Kernel length	*hKL1a*	1.02	umc2191-bnlg1007-bnlg1083	5.67	0.009
		*hKL3b*	3.07	umc1489-umc1825-phi046	−4.33	0.041
		*hKL5b*	5.09	umc1792-umc1153	−7.33	0.005
		*hKL6c*	6.04	umc1979-nc009-umc1014	−7.50	0.027
		*hKL7a*	7.02	umc1666-umc1703-umc1433	−10.00	0.017
		*hKL9a*	9.01	umc1809-umc2093-umc1170	−7.00	0.045
		*hKL9b*	9.06	bnlg1091-bnlg1191-umc2345	−8.00	0.013
	Kernel width	*hKW1a*	1.03	umc1403-umc1397-bnlg182	−13.74	0.04
		*hKW3a*	3.05	umc1954-umc2166-umc1593	−6.04	0.026
		*hKW3d*	3.08	phi046-umc1844-umc2275	−4.95	0.044
		*hKW3e*	3.09	umc1320-umc2152-umc2277	−10.99	0.007
		*hKW4*	4.03	umc1757-umc2280-umc1550	−13.55	0.016
		*hKW6c*	6.06	bnlg1732-umc1424-umc1296	7.69	0.038
		*hKW7b*	7.03	bnlg2271-umc1112-bnlg1805	−8.79	0.027
		*hKW7d*	7.04	umc2332-phi328175-umc1295	6.23	0.006
		*hKW9b*	9.05	umc1519-umc1375-umc1231	4.40	0.026
		*hKW9c*	9.06	bnlg1091-bnlg1191-umc2345	−7.69	0.012
	Kernel thickness	*hKT1a*	1.04	bnlg182-bnlg2238-umc1144	−4.64	0.001
		*hKT1b*	1.07	umc1356-umc1278-umc1013	4.87	0.001
		*hKT3f*	3.09	umc1320-umc2152-umc2277	4.35	0.005
		*hKT6a*	6.03	umc1178-phi389203-umc2316	8.17	0.001
		*hKT6b*	6.04	umc1979-nc009-umc1014	7.28	0.002
		*hKT6c*	6.05	mmc0523-umc2006-umc1614	6.66	0.004
		*hKT6d*	6.06	bnlg1732-umc1424-umc1296	3.52	0.002
		*hKT6e*	6.07	umc2165-bnlg1136-umc1653	7.30	0.000
		*hKT7a*	7.02	bnlg1792-umc1929-umc1585	2.35	0.003
		*hKT7c*	7.04	bnlg1805-umc2332-phi328175	2.32	0.001
		*hKT7d*	7.04	bnlg2271-umc1112-bnlg1805	2.73	0.003
		*hKT9b*	9.02	umc1170-umc1037-umc1033	5.90	0.002
		*hKT9e*	9.06	bnlg1191-umc2345-umc1310	−3.98	0.000
	100-kernel weight	*hHKW1a*	1.01	bnlg1179-umc1269-umc2012	21.36	0.007
		*hHKW1d*	1.08	umc1278-umc1013-bnlg2228	21.14	0.001
		*hHKW2a*	2.03	bnlg1792-umc1929-umc1585	−18.00	0.012
		*hHKW3a*	3.04	umc2259-phi036-umc1495	13.48	0.006
		*hHKW3e*	3.07	umc2050-umc1135-umc1399	15.01	0.046
		*hHKW5b*	5.06	phi085-phi048-umc2201	10.2	0.008
		*hHKW5b*	5.06	bnlg278-umc1680-phi085	7.58	0.041
Hebi	Kernel length	*hKL1b*	1.03	umc1397-bnlg182-bnlg2238	3.53	0.008
		*hKL1b*	1.03	umc1403-umc1397-bnlg182	3.33	0.021
		*hKL1c*	1.06	umc1281-umc2151-umc1035	−4.89	0.002
		*hKL9a*	9.01	umc1809-umc2093-umc1170	−3.17	0.040
		*hKL9a*	9.02	umc1170-umc1037-umc1033	−5.51	0.003
	Kernel width	*hKW3c*	3.05	umc2127-umc1954-umc2166	−10.6	0.028
		*hKW3d*	3.08	phi046-umc1844-umc2275	−6.44	0.043
		*hKW7a*	7.02	bnlg1792-umc1929-umc1585	−11.6	0.026
		*hKW9a*	9.02	umc1037-umc1033-bnlg1082	6.55	0.037
		*hKW9b*	9.05	umc1492-umc1519-umc1375	8.21	0.025
	Kernel thickness	*hKT1b*	1.07	umc1335-umc2396-umc1356	3.64	0.013
		*hKT1c*	1.08	umc1013-bnlg2228-dupssr12	5.86	0.024
		*hKT1d*	1.11	umc2047-umc1538-bnlg131	−5.87	0.005
		*hKT3e*	3.07	umc1135-umc1399-umc1148	7.31	0.050
		*hKT4a*	4.01	umc1228-umc1017-umc1757	4.42	0.003
		*hKT4a*	4.01	phi072-umc1228-umc1017	6.68	0.016
		*hKT4c*	4.09	umc2211-umc1101-bnlg589	5.41	0.000
		*hKT6b*	6.04	umc1979-nc009-umc1014	6.60	0.033
		*hKT9a*	9.01	umc1809-umc2093-umc1170	6.81	0.012
		*hKT9d*	9.05	umc1231-umc1494-bnlg1091	−5.48	0.039
	100-kernel weight	*hHKW1b*	1.04	bnlg182-bnlg2238-umc1144	5.12	0.035
		*hHKW2c*	2.04	bnlg1064-umc1024-umc1465	−4.80	0.033
		*hHKW3c*	3.04	umc1908-umc1773-phi053	5.88	0.029
		*hHKW3d*	3.05	umc2127-umc1954-umc2166	11.59	0.050
		*hHKW5a*	5.01	bnlg1006-phi024-bnlg1879	6.74	0.000
		*hHKW6*	6.05	umc1614-umc2141-umc1805	5.77	0.047
		*hHKW7b*	7.03	umc1567-bnlg1305-bnlg2271	6.49	0.013
		*hHKW7c*	7.04	umc2332-phi328175-umc1295	13.21	0.015
		*hHKW9c*	9.02	umc1037-umc1033-bnlg1082	10.99	0.025

For kernel width, 13 different HL were identified in the CSSLs × Xun9058 population, of which only *hKW9b* was detected at the two locations simultaneously. This contributed 4.40% and 8.21% to kernel width compared with the control hybrid at Changge and Hebi, respectively.

Twenty HL for kernel thickness were detected in the CSSLs × Xun9058 population. *hKT1b*, identified at both locations simultaneously, increased kernel thickness by 4.87% and 3.64% compared with the control hybrid at Changge and Hebi, respectively, while *hKT6b* made 7.28% and 6.60% contributions compared with the control hybrid at the same locations.

Fifteen different HL for 100-kernel weight were identified in the CSSLs × Xun9058 population at the two locations, including 10 detected at Changge and eight at Hebi.

### Comparison of heterotic loci identified in the two test populations

When comparing common HL detected at the two test populations, only 21 (33.33% and 36.84%) for the four kernel traits were detected in this study simultaneously (Supplemental Table 1); most HL (42/63, 66.67%; 36/57, 63.16%) differed between the two test populations. This supports the notion that heterosis is controlled by multiple loci, and that the interaction of multiple loci affects heterosis for a given trait in different hybrids. The HL detected in the two test populations simultaneously, such as *hKL9b*, *hKW9a*, *hHKW1d*, *hHKW3a*, and *hHKW7b*, may be common for the measured traits between the Reid × Tangsipingtou (TSPT) heterotic pattern, so could be used to predict hybrid performance in future maize breeding.

## Discussion

### The use of CSSL test populations with different parents

Because parental lines of maize commercial hybrids belong to different heterotic groups, the HL involved in different hybrids are always diverse. hQTL and HL mapping research has usually been carried out with biparental populations such as F_2:3_, DH, and RIL test populations^13,18,38–40^, IF_2_ populations^2,16^, and CSSLs or SSSLs test populations^23,37,41,42^. Although each type of segregating population has its own merits and corresponding shortcomings, none of them can be used to compare HL derived from different crosses. Larièpe *et al*.^43^ adopted an extension of design III derived from three initial inbred parents. The NCIII extension enabled the study of heterosis in families derived from both unrelated and related parents, and comparisons of contrasts between homozygous and heterozygous genotypes and between heterozygous genotypes to be made at each locus.

In this study, out of the 56 QTL and 120 HL for kernel traits identified using a CSSL population and its two test populations, only four QTL and HL (*qKT1 vs hKT1c*, *qKT1a vs hKT1b*, *qKT9b vs hKT9b*, *qHKW9b vs hHKW9c*) were detected at the same or overlapping introgression region lines at the same locations (Tables 4–6); many QTL (52/56, 92.86%) and HL (117/120, 97.50%) were not located on the same chromosomal region. This result indicated that heterosis and performance are controlled by different genetic mechanisms^37^, and that the HL for kernel construction traits detected by comparing each CSSL test hybrid with its corresponding CK have different genetic effects in the inbred lines lx9801 and Chang7-2 compared with the two test parents. Additionally, the CSSL population was tested further with multiple inbred lines belonging to the Reid heterotic group to identify HL between Reid and TSPT heterotic group. This type of test population can therefore not only identify specific HL of multiple inbred lines but can also be used to screen for common HL between Reid × TSPT heterotic model systems.

### The advantage of heterotic loci identification using CSSLs test populations

Heterosis is a complicated characteristic controlled by minor additive and dominant multigenes. It is also readily affected by environmental factors such as soil fertility, sunlight, rainfall, and plant density^44^. Several important factors have limited dissection of the heterosis genetic basis. First, many agronomic traits and economical characters are compound traits involving several secondary traits, so trait heterotic values are the concurrent results of secondary traits. Second, it is debatable whether mid-parent heterosis or over-parent heterosis data represent the real heterotic expression, although mid-parent heterosis has been previously used to identify heterotic loci^16,21^. Third, the accuracy of phenotypic values of heterosis in different environments is uncertain when using mid-parent heterosis to dissect the genetic basis of heterosis. This is because inbred lines are easily affected by environmental factors, so mid-parent heterosis values vary in different environments. Finally, because the heterotic gene always has a distinctly different genetic effect among different heterozygous alleles, it is important to identify common heterotic loci between different test parents.

In previous studies, several types of segregated populations have been used to dissect the genetic basis of heterosis; these include F_2_ populations, DH and RIL test populations, IF_2_ populations, triple testcross populations, and SSSL backcross populations^3,14,16,17,38,45–49^. In comparison with these, the CSSLs test populations with different test parents in this study has several merits for dissecting the genetic basis of heterosis. First, the heterotic loci identified by comparing the test population and its corresponding CK have little influence in different environments, so it is easy to obtain accurate phenotypic values. Second, the genetic effect of heterotic loci in different test backgrounds can be analysed to identify the main heterotic loci between different groups. Theoretically, if the significant loci identified by comparing test hybrids and corresponding CK are true HL, many will not be simultaneously detected in the CSSL population. In this study, only four QTL and HL were identified simultaneously in the same CSSLs, and most HL (97.50%) were not detected in the CSSL population. Thus, the significant loci identified were real HL, which showed different heterotic genetic effects between the two inbred lines lx9801 and Chang7-2 when tested against the inbred Zheng 58 and Xun 9058.

In a previous study, Wang *et al*.^50^ identified 169 HL associated with grain yield and its five components using two test populations. Additionally, we identified several common HL of kernel-related traits over the same and overlapping introgression region lines. These included *hKW3d* for kernel width, *hHKW3b* and *hHKW5b* for 100-kernel weight, *hKL1a* and *hKL9a* for kernel length, and *hKT4a* for kernel thickness in the CSSLs × Zheng58 population (Tables 5, 6).

### Hybrid performance production

The identification of high-performing hybrids is an integral part of every maize breeding program. However, because the field evaluation of all potential hybrids is too resource-intensive, only a small subset are tested in field trials, from which only a few elite hybrids are selected^51^. Therefore, it is important to estimate hybrid performance^52^. In a previous study, hybrid vigour was predicted using molecular markers to estimate genetic distances among parents^53^; several studies have uncovered a direct correlation between superior hybrid performance and the genetic distance of parental lines^54,55^. Recent investigations have used molecular markers and QTL for the genomic prediction of hybrid performance in maize^56–58^, sunflowers^8^, and wheat^26^. An important component of hybrid performance is the specific combining ability between the parental lines of a hybrid. As a consequence, both additive and dominance effects of markers must be estimated to account for the entire genetic variance.

Using a simulation study, Technow *et al*.^51^ demonstrated a strong alternative to genomic best linear unbiased prediction, in the form of the Bayesian whole-genome regression method, BayesB^59^. This method was first used by Maenhout *et al*.^57^ for the genomic estimation of hybrid performance. In terms of heterosis optimum manipulation, the parental inbred lines of different hybrids have been extracted from genetically distant germplasm pools, called heterotic groups^53^, and have been widely used by maize breeders. As for hybrid prediction, a key question is how many hybrids per inbred line, i.e., a cross with lines from the opposite heterotic group, should be included in the training set. Technow *et al*.^58^ followed the Dent and Flint heterotic pattern to analyse the genomic and phenotypic data of 1,254 hybrids in a typical maize hybrid breeding program. They found that the estimated accuracy of untested hybrids was highest if both parents were parents of other hybrids in the training set, and that the prediction accuracy of untested hybrids was lowest if neither parents were involved in any training set hybrid. Technow *et al*.^51^ also showed that prediction accuracies increased with marker density and the number of tested parents. They reported that under low linkage disequilibrium the modelling of marker effects as population-specific was the most beneficial. In China, Reid and TSPT are components of the first heterotic pattern that has been widely used in maize breeding^60^. In the present study, two test populations, constructed from representative inbred lines derived from the Reid and TSPT heterotic group, were used to detect HL for kernel traits in maize. We detected 21 HL in the two test populations simultaneously, and suggest that these HL for kernel traits and their linked molecular markers could be used to predict hybrid performance in future maize breeding programs.

### Genetic dissection of heterosis for kernel traits in maize

Grain yield is the ultimate product of multiple processes that occur throughout the growing season^61^. Grain yield in maize is a function of many harvested kernels and their individual weights. In these two components, the number of kernels usually explains most of the variation^62^, while kernel weight has a high heritability^63,64^ and varies significantly among genotypes^65^. Several QTL mapping studies for maize kernel weight have been conducted, but results are inconsistent in terms of effect size and localisation^32–34^. Such inconsistencies may be linked to the complexity of the trait, which thus requires further dissection into simpler components. Regarding the secondary traits of kernel weight, Zhang *et al*.^35^ detected three QTL for kernel depth using a 229-line F_2:3_ population derived from inbred lines Yu 82 and Shen 137. Zhang *et al*.^36^ also identified 54 unconditional main QTL for five kernel-related traits, namely kernel thickness, weight, length, volume, and width using an IF_2_ population in maize. In the present study, 63 HL for four kernel traits were identified in the CSSLs × Zheng58 population, with nine (13.8%), namely *hKL9a*, *hKW*2, *hKW9b*, *hKT1d*, *hKT2a*, *hHKW1d*, *hHKW3a*, *hHKW3b*, and *hHKW7b*, identified at two locations simultaneously. Fifty-seven HL were identified for four kernel traits in the CSSLs × Xun9058 population, of which seven (12.18%), *hKL9a*, *hKW3c*, *hKW3d*, *hKW7a*, *hKW9b*, *hKT1b*, and *hKT6b*, were identified for four kernel traits at two locations simultaneously. Several HL for different kernel traits sharing common chromosomal regions were also detected. For example, HL for 100-kernel weight, length, thickness, and width were detected in chromosomal bin 2.03 in the same chromosomal region, umc2195-umc1555-bnlg1064, in the CSSLs × Zheng58 population at the two locations simultaneously. Moreover, the HL for kernel length and thickness were detected in chromosomal bin 9.02 at the same chromosomal region, bnlg1170-umc1037-umc1033, in the CSSLs × Xun9058 population at the two locations.

Many HL detected for grain yield and its components in a previous study^50^, as well as HL for kernel traits located on common chromosomal regions in the same test populations have been found on the same chromosomal region. For example, in the CSSLs × Xun9058 population, HL *hlEL1a* for ear length, *hlEW1b* for ear width, *hlGY1a* for grain yield, and *hKW1a* for kernel width were detected on umc1403-umc1397-bnlg182. Similarly, HL *hlEL1b* for ear length, *hlRN1a* for row number, *hlKPR1b* for kernels per row, *hKT1a* for kernel thickness, and *hHKW1b* for 100-kernel weight were detected on the chromosomal region bnlg182-bnlg2238-umc1144, while HL *hlEW9b* for ear width, *hlRN9c* for row number, *hlKPR9b* for kernels per row, *hKT9b* for kernel thickness, and *hKL9a* for kernel length were located on umc1170-umc1037-umc1033. In the CSSLs × Zheng58 population, HL *hlEL1b* for ear length, *hlRN1a* for row number, *hlGY1b* for grain yield, *hKW1b* for kernel width, *hHKW1b* for 100-kernel weight, and *hKT1a* for kernel thickness were found on bnlg182-bnlg2238-umc1144. Moreover, on the chromosomal region umc1844-umc2275-umc2081, HL *hlEL3e* for ear length, *hlEW3e* for ear width, *hlRN3e* for row number, *hKL3d* for kernel length, *hKW3d* for kernel width, and *hHKW3f* for 100-kernel weight were detected simultaneously. These results show that the HL for grain yield and its components form clusters, and that the regions containing these clusters may be useful for future maize breeding.

### Combining ability and heterosis in maize

It can be difficult to predict the yield performance of hybrids by that of their parents *per se* in breeding practice^66^. Therefore, selecting inbred lines with a high combining ability becomes important. Griffing^67^ first carried out diallel tests to comprehend the genetic basis of combining ability, and proposed that SCA and GCA were mainly respectively correlated with nonadditive and additive genetic effects. In a previous study, Qi *et al*.^68^ identified 56 significant loci for GCA and 21 loci for SCA of five yield-related traits of introgression lines test populations in maize, and only five loci for GCA and SCA simultaneously; this indicated a different genetic basis for GCA and SCA.

Significant positive correlations were also reported for SCA with high parent heterosis (HPH), mid parent heterosis (MPH), and hybrid performance^69,70^. Hence, improved selection for SCA would indirectly increase MPH and HPH for hybrids in maize breeding^70^. In this study, 63 and 57 different HL were identified for four kernel traits in the CSSLs × Zheng58 and CSSLs × Xun9058 populations, of which only 21 (33.33% and 36.84%) were detected in the two test populations simultaneously. The results are consistent with different SCA for different crosses in previous studies^69–71^. Moreover, our work suggests that common HL identified from different tests can be used to select elite inbred lines with high SCA, and contribute to predicting hybrid performance in the Reid × TSPT heterotic model in maize breeding.

## Materials and Methods

### CSSL and test population construction and field experiments

A maize 184 CSSL population constructed using two elite inbred lines, lx9801 and Chang7-2, was used in this study. The two elite inbred lines belonged to the TSPT heterotic group, an important germplasm widely used in China. The inbred line Chang7-2, used as the donor parent, is one parent of the elite hybrid Zhengdan958 (Zheng58 × Chang7-2), the first commercial hybrid widely used in China (from 2005 to 2014). The recipient parent was lx9801, a parent of Ludan9002 (Zheng58 × lx9801), another elite commercial hybrid. The two hybrids, Zhengdan958 and Ludan9002, have a common female parent, Zheng58. Based on the simple sequence repeat (SSR) molecular marker linkage map integrated by IBM 2008 Neighbors (http://www.maizegdb.org), 700 paired SSR markers were used to screen for polymorphisms between the two parents, and a total of 225 SSR polymorphic markers were used to screen donor fragments of the inbred line Chang7-2 at different backcross populations.

In the winter of 2009, 929 BC_3_F_1_ lines were planted in Sanya (N18°15′, E109°30′) China. Five plants of each BC_3_F_1_ line were screened using the polymorphic markers, and 875 plants with fewer (<5) donor chromosomal regions were backcrossed with recipient parents to produce the BC_4_F_1_ population. BC_4_F_1_ plants with one or two donor chromosomal regions were screened using polymorphic markers, and selfed in the summer of 2010 in Zhengzhou (N34°16′, E112°42′) China. In the winter of 2011, 1718 BC_4_F_2_ lines were selected and planted in Sanya, and plants with single chromosomal segment substitutions were verified and selfed to produce 102 homozygous CSSLs. Additionally, some single heterozygous CSSLs were also selfed, and 84 were selected from the BC_4_F_3_ population. Finally, we achieved a population of 184 CSSLs with a single segment of donor parent Chang7-2 in the recipient parent lx9801 background^50^.

**Figure 3:**
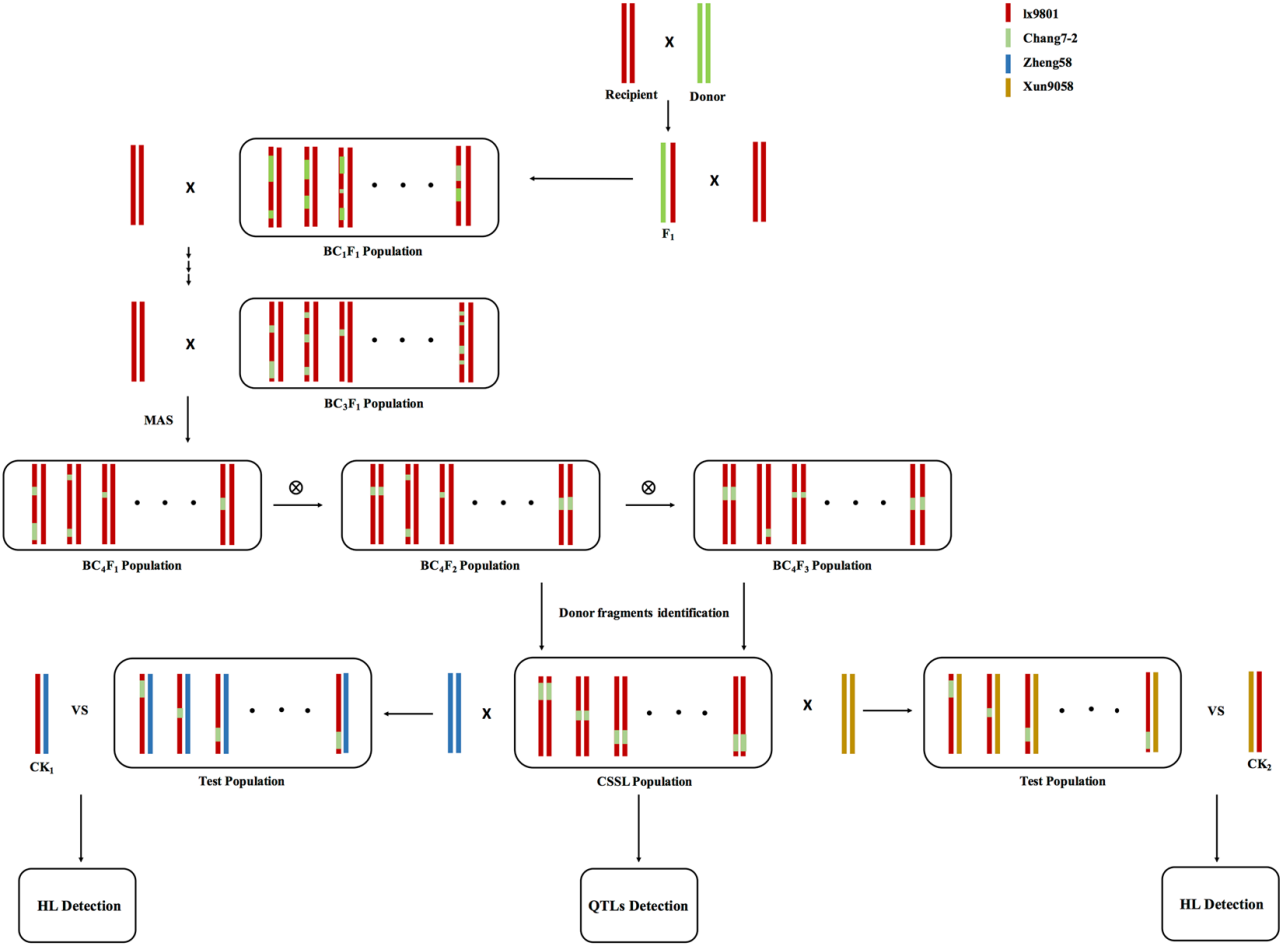
Group construction pattern map. Each genome is represented by a specific color. Red and green represents lx9801 as recipient parent and Chang7-2 as donor parent respectively; Blue and brown represents test parent Zheng58 and Xun9058. HL, heterotic loci; QTLs, quantitative traits loci; CK1, Zheng58 × lx9801; CK2, Xun9058 × lx9801; MAS, marker-assisted selection.

Test populations were constructed using the CSSL population and two inbred lines, Zheng58 and Xun9058 (Fig. 3). These inbred lines belong to the Reid heterotic group, which are a pair of the heterotic model Reid × TSPT, and are widely used in China.

### Field experiments

Because test hybrids are taller than the CSSL population, the planting of test hybrids adjacent to their parents in the field would affect the normal growth of inbred lines. To avoid interactions of test hybrids and the CSSL population, they were divided into two individual experiments in this study. First, the two test populations and their corresponding control hybrids (Zheng58 × lx9081 and Xun9058 × lx9081) were evaluated on farms of the Hebi Agricultural Institute (Hebi; E 114° 33′, N 35° 41′) and Changge (E 113° 29′, N 34° 1′), respectively. Materials were planted in June 2012 and 2013 following the wheat harvest. The experimental design of the test population consisted of a randomised complete block with three replicates of corresponding hybrids (Zheng58 × lx9801 and Xun9058 × lx9801; CK) added between the 10 test crosses. Each plant material was planted in one plot per field. The plots constituted of rows 4 m long, separated by 0.66 m between each row. A total population density of 67,500 plants per hectare was maintained. The CSSL population and the four inbred lines (lx9801, Chang7-2, Zheng58, and Xun9058) were planted in the adjacent field using the same field design to identify the additive QTL and analyse mid-parent heterosis. Fields were managed according to local maize cultivation practices.

### Performance measurement

After maturity, 10 ears were harvested from successive plants in each plot, and air-dried to a grain moisture level of 13%. Measured traits were kernel length (mm), kernel thickness (mm), kernel width (mm), and 100-kernel weight (g). Phenotypic data were recorded as follows: (1) KL (mm kernel^−1^) = (ear diameter − cob diameter)/2, for which cob and ear diameters were measured at the middle of the ear; (2) kernel width in the middle of a kernel (KW, mm kernel^−1^) = [cob diameter + (ear diameter − cob diameter)/2] π/(ear row number); (3) KT (mm kernel^−1^), judged from the thickness of 10 kernels in the middle of an ear^36,72,73^; (4) HKW (g), the average value of the three measurements of the weight of 100-kernels randomly selected^74^.

### Data analysis

Mid-parent heterosis (H_MP_) for the four kernel traits in the two test populations was evaluated in the two environments. The values of mid-parent heterosis were calculated as H_MP_ (%) = (F_1_ − MP)/MP × 100%, for which H_MP_ is the percentage of mid-parent heterosis^75^, F_1_ is the average data of the four kernel traits in each testcross population, and MP is the mean of the average values of the CSSL population and the corresponding test parent. Mid-parent heterosis values of the corresponding hybrids (Zheng58 × lx9801 and Xun9058 × lx9801) were calculated using the same formula.

SAS 9.2 was used to evaluate the four kernel traits of the two test populations exhibited significant variation between locations and genotypes. The model followed was: phenotype_ijk_ = μ + location_i_ + genotype_j_ + genotype*location + repetition_k_ + error_ijk_, where μ is overall mean^76^. H^2^_B_ = σ^2^_genotype_/(σ^2^_genotype_ + σ^2^_genotype*location_/e + σ^2^_error_/r*e), where e and r are the numbers of environments and replicates^76^.

A QTL was considered to exist in the CSSL population when there was a significant difference in the measured value between the CSSL and the recurrent inbred line lx9801 (*P* < 0.05) by one-way analysis of variance and Duncan’s multiple comparisons using SPSS 17.0 software. The additive effect was calculated using the following equation: A = (SSSL − lx9801)/2. The percentage of the additive effect (A%) was calculated using the following equation^2^: A% = A/lx9801 × 100%.

Heterotic genes usually have different genetic effects and phenotypes when heterozygosis with different alleles is observed^50^. We identified HL for kernel-related traits and compared the significance between single hybrids and the average value of two adjacent corresponding CK (hybrids Zheng58 × lx9801 or Xun9058 × lx9801). The value of a given trait of one test hybrid in the three replicates between two years at one location was compared between the test population and its corresponding hybrid using the Student’s t-test. If a significant difference was observed (*P* < 0.05), the corresponding chromosomal region was considered to be a HL between the CSSL and its test inbred line, and the HL showed a different heterotic genetic effect between inbred line Chang7-2 and lx9801. The heterotic effect was calculated as follows: HL% = (H − CK)/CK × 100%, where H represented the value of the trait in a single cross in the CSSL test populations and CK represented the value of the trait in the corresponding hybrid^77^.

## Electronic supplementary material

Supplemental table 1
