# Supplementary material for: Heterotic loci identified for maize kernel traits in two chromosome segment substitution line test populations
Source: Sci Rep. 2018 Jul 23;8:11101. doi: 10.1038/s41598-018-29338-1 (PMC6056474; doi:10.1038/s41598-018-29338-1)
Supplement: Supplementary file 1 — Supplemental table 1 [file 41598_2018_29338_MOESM1_ESM.doc]

**Supplemental table 1 for**

**Heterotic loci identified for maize kernel traits in two chromosome segment substitution line test populations**

Yafei Wang^1#^, Xiangge Zhang^1,2#^, Xia Shi^1^, Canran Sun^1^, Jiao Jin^1^, Runmiao Tian^1^,

Xiaoyi Wei^3^, Huiling Xie^1^, Zhanyong Guo^1*^ & Jihua Tang^1,4*^

1. National Key Laboratory of Wheat and Maize Crop Science, Henan Agricultural University, Zhengzhou 450002, China
2. Agronomy College, Sichuan Agricultural University, Wenjiang 611130, China

3 Xinxiang Academy of Agricultural Sciences, Xinxiang 453003, China

4 Hubei Collaborative Innovation Centre for Grain Industry, Yangtze University,

Jingzhou 434025, China

**Supplemental table 1.** Common heterotic loci detected for kernel-related traits in the CSSLs × Zheng58 and CSSLs × Xun9058 populations.

| Traits | HL | CSSLs × Zheng58 | | CSSLs × Xun9058 | |
| --- | --- | --- | --- | --- | --- |
|  |  | Changge | Hebi | Changge | Hebi |
| Kernel length | *hKL1b* | + |  | + |  |
|  | *hKL1c* | + |  |  | + |
|  | *hKL9a* |  |  | + | + |
|  | *hKL9b* | + | + | + |  |
| Kernel Width | *hKW1a* |  | + | + |  |
|  | *hKW2* | + | + |  |  |
|  | *hKW3a* | + |  | + |  |
|  | *hKW3c* |  |  | + | + |
|  | *hKW3d* | + |  | + | + |
|  | *hKW7a* | + |  | + | + |
|  | *hKW7d* | + |  | + |  |
|  | *hKW9a* | + | + |  | + |
|  | *hKW9b* |  |  | + | + |
| Kernel thickness | *hKT1a* |  | + | + |  |
|  | *hKT1b* |  |  | + | + |
|  | *hKT1c* | + |  | + |  |
|  | *hKT1d* | + | + |  | + |
|  | *hKT2a* | + | + |  |  |
|  | *hKT6b* |  |  | + | + |
|  | *hKT6e* |  | + | + |  |
|  | *hKT9a* |  | + |  | + |
|  | *hKT9b* |  | + | + |  |
| 100-kernel weight | *hHKW1b* | + |  |  | + |
|  | *hHKW1d* | + | + | + |  |
|  | *hHKW3a* | + | + | + |  |
|  | *hHKW3b* | + | + |  |  |
|  | *hHKW3d* | + |  |  | + |
|  | *hHKW7b* | + | + | + |  |
|  | *hHKW9c* |  | + |  | + |

HL, heterotic loci
